# Pharmacological resynchronisation with flecainide in an infant with Ebstein anomaly and Wolff-Parkinson-White pattern: a case report

**DOI:** 10.1093/ehjcr/ytae442

**Published:** 2024-09-02

**Authors:** Gajon Uthayakumaran, Hiroko Asakai, Ganesh Gnanappa

**Affiliations:** The Heart Centre for Children, The Children’s Hospital at Westmead, Corner Hawkesbury Road and Hainsworth St, Westmead, NSW 2145, Australia; The Heart Centre for Children, The Children’s Hospital at Westmead, Corner Hawkesbury Road and Hainsworth St, Westmead, NSW 2145, Australia; The Heart Centre for Children, The Children’s Hospital at Westmead, Corner Hawkesbury Road and Hainsworth St, Westmead, NSW 2145, Australia

**Keywords:** Ebstein anomaly, Wolff-Parkinson-White pattern, Left ventricular dysfunction, Case report

## Abstract

**Background:**

Conduction abnormalities are frequently encountered in patients with Ebstein anomaly. The following case describes the safe use of flecainide in an infant with accessory-pathway mediated left ventricular dysfunction in the setting of Ebstein anomaly.

**Case Summary:**

An infant with an antenatal diagnosis of Ebstein anomaly developed progressive left ventricular dilatation and dysfunction over the first 2 months of life. ECG demonstrated persistent Wolff-Parkinson-White pattern with delta-wave polarity suggesting a right-sided septal accessory pathway. In the absence of SVT, accessory-pathway mediated dyssynchrony was suspected as the cause for left ventricular dilatation and dysfunction. He was commenced on flecainide which successfully blocked antegrade conduction via the accessory pathway resulting in a reduction in left ventricular volume and improvement in left ventricular systolic function. He remains asymptomatic at 12 months of age.

**Discussion:**

There is a known association between Ebstein anomaly and Wolff-Parkinson-White pattern. Right-sided septal accessory pathways can cause cardiomyopathy secondary to dyssynchronous left ventricular contraction. In patients who are unsuitable for accessory pathway ablation, flecainide can be used to block antegrade conduction via the accessory pathway resulting in improved left ventricular function, which was successful on this occasion.

**Learning points**

- In patients with Ebstein anomaly and left ventricular dysfunction, consider dyssynchrony due to accessory-pathway mediated conduction as a cause.
- Flecainide can be safely used in selected infants with Ebstein anomaly and ventricular pre-excitation to achieve resynchronisation and normalisation of left ventricular size and systolic function.

## Introduction

Ebstein anomaly is a rare congenital cardiac anomaly, which occurs due to incomplete delamination of the tricuspid valve from the right ventricular endocardium. There is a well-established association between Ebstein anomaly and a broad range of arrhythmia phenotypes. These include atrial arrhythmias such as ectopic atrial tachycardia and atrial flutter, supraventricular tachycardia (SVT) with or without Wolff-Parkinson-White pattern, and rarely ventricular arrhythmias.^1^ We report a case in which pharmacological resynchronisation was achieved using flecainide in an infant with accessory pathway-mediated left ventricular dysfunction in the setting of Ebstein anomaly.

## Summary figure

**Figure:**
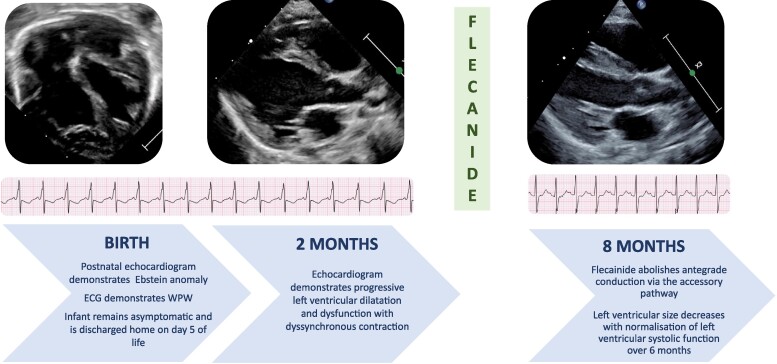


## Case report

**Figure 1:**
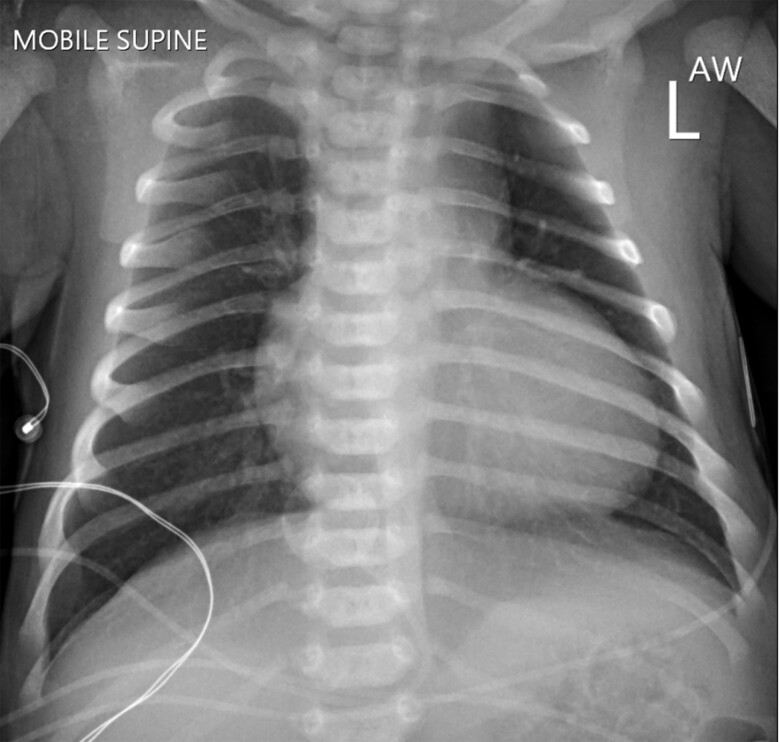
Chest x-ray on day 2 of life demonstrating mild cardiomegaly and no other abnormalities.

**Figure 2:**
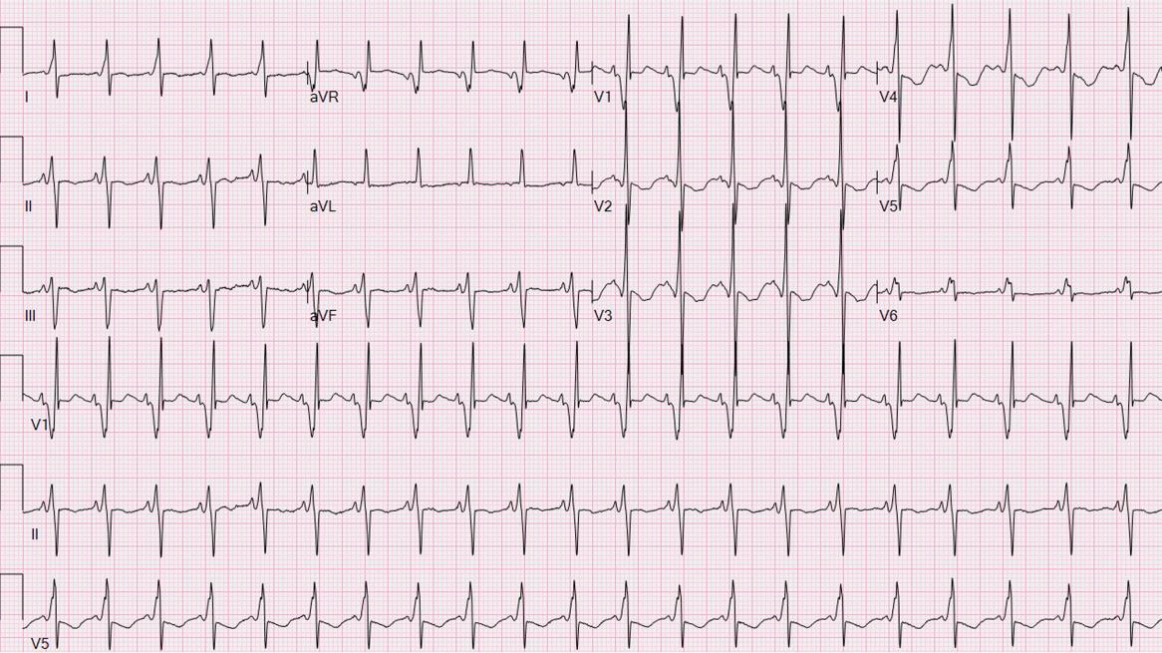
Twelve-lead ECG on day 2 of life demonstrating Wolff-Parkinson-White pattern.

A male newborn was delivered at 40^+3^ weeks gestation via a normal vaginal delivery with a birthweight of 3.2 kg. There was an antenatal diagnosis of Ebstein anomaly and foetal echocardiogram at 35^+6^ weeks demonstrated no significant tricuspid regurgitation with normal antegrade flow across the pulmonary valve and normal right ventricular function. There were no maternal medications and the antenatal history was otherwise unremarkable. The baby was delivered in good condition with APGARs of 8^1^ and 9^5^. There was no requirement for respiratory support and oxygen saturations were >90% in room air. He was transferred to the neonatal intensive care unit for observation and a post-natal echocardiogram on day 1 of life confirmed a diagnosis of Ebstein anomaly with mild tricuspid regurgitation. There was an associated patent foramen ovale with right-to-left shunt and normal antegrade pulmonary blood flow. There was a moderate patent ductus arteriosus on day 1 of life with left-to-right shunt. The left ventricle was mildly dilated with dyssynchronous contraction of the interventricular septum and mild systolic dysfunction (see Supplementary material online, *Video S1*). A chest x-ray on day 2 of life demonstrated mild cardiomegaly with no other abnormalities (*Figure 1*). A 12-lead ECG on day 2 of life demonstrated sinus rhythm with left axis deviation, right atrial enlargement and Wolff-Parkinson White pattern (*Figure 2*). Delta-wave polarity suggested a right-sided accessory pathway. He remained stable with normal oxygen saturations and feeds were established without complication. There were no documented episodes of SVT. He was discharged home on day 5 of life with outpatient follow-up.

**Figure 3:**
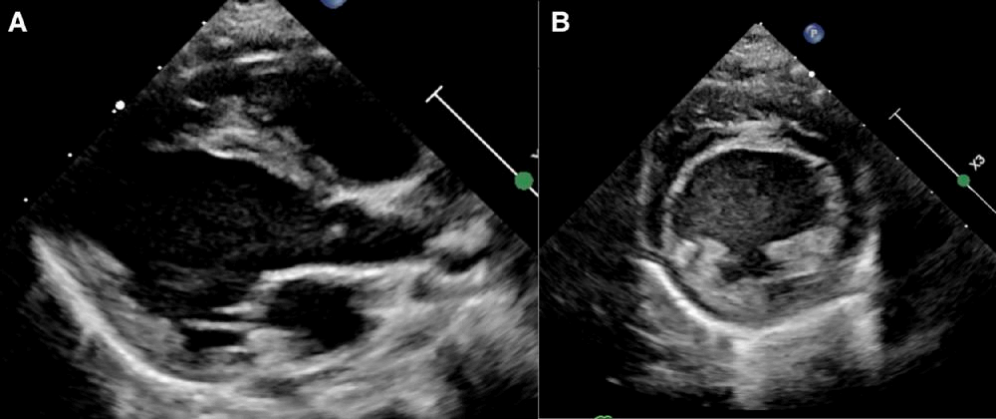
Two-dimensional parasternal long-axis (*A*) and short-axis (*B*) images at end-diastole demonstrating moderate left ventricular dilatation.

Serial outpatient follow-up was organised in early infancy and the baby remained asymptomatic from a cardiac perspective. A repeat echocardiogram at 2 months of age demonstrated mild tricuspid regurgitation. The degree of tricuspid regurgitation and right ventricular size were unchanged, however the left ventricle had progressively dilated (left ventricular end-diastolic diameter *Z*-score +4.6) with associated moderate systolic dysfunction (*Figure 3*, Supplementary material online, *Video S2*). As noted previously, there was significant dyssynchronous contraction of the interventricular septum. There was also mild-moderate mitral regurgitation, which had progressed as the initial echocardiogram on day 1 of life. There was no evidence of left ventricular non-compaction. The ECG was unchanged from previous and a Holter monitor was performed which demonstrated persistent WPW pattern with no episodes of SVT. He was also referred for genetic testing to exclude a co-existent cardiomyopathy.

**Figure 4:**
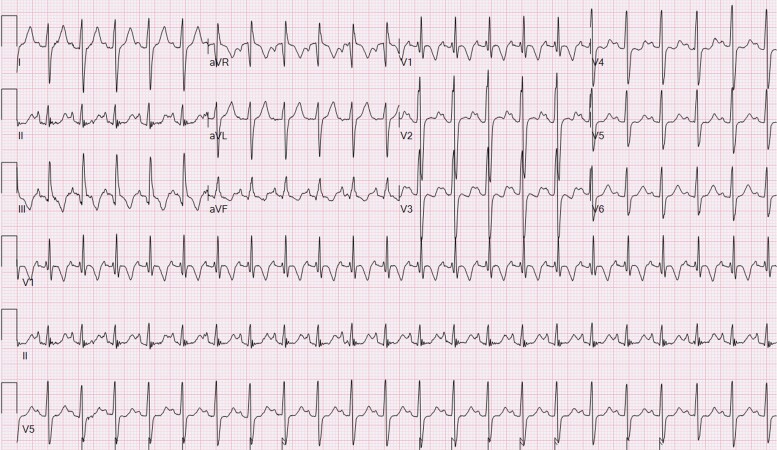
Twelve-lead ECG at 2 months of life after commencement of Flecainide demonstrating sinus rhythm with first-degree atrioventricular block and abolishment of ventricular pre-excitation.

**Figure 5:**
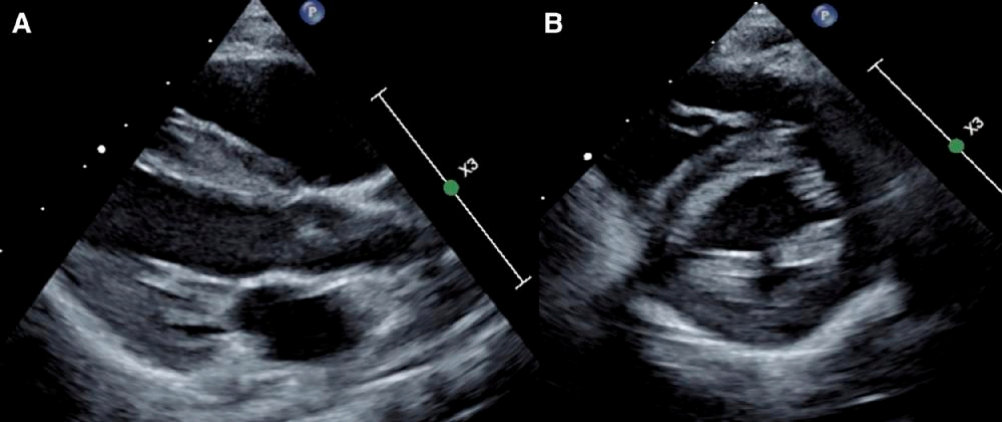
Two-dimensional parasternal long-axis (*A*) and short-axis (*B*) images at end-diastole demonstrating reduced left ventricular size after antegrade conduction via the accessory pathway was abolished with flecainide.

In view of the dyssynchrony, the infant was commenced on oral flecainide at a dose of 2 mg/kg/dose twice daily, which successfully blocked antegrade conduction via the accessory pathway (*Figure 4*). Over the following 8 months, the baby received outpatient follow-up on a 2-monthly basis. Serial echocardiography demonstrated ongoing dyssynchronous contraction of the interventricular septum; however, there was a gradual reduction in left ventricular size with normalisation of systolic function and resolution of mitral regurgitation. The degree of tricuspid regurgitation and right ventricular dilatation has remained stable (*Figure 5*, Supplementary material online, *Video S3*). The infant remains on oral flecainide at a dose of 2 mg/kg/dose twice daily and there have been no symptoms reported throughout the follow-up period. There is no plan for intervention and clinical surveillance continues.

## Discussion

Ebstein anomaly is a rare congenital cardiac anomaly with an estimated incidence of ∼1 in 200 000 livebirths, accounting for <1% of all congenial heart disease.^2^ There is a broad anatomic and clinical spectrum of disease with presentations varying from intrauterine foetal demise to incidental detection in adulthood. Electrocardiographic abnormalities are common in Ebstein anomaly with typical findings including right atrial enlargement, first-degree atrioventricular block and right bundle branch block.^2^ Accessory atrioventricular pathways are seen in up to 30% of patients with Ebstein anomaly.^3^ The substrate for accessory pathways in Ebstein anomaly is related to the downward displacement of the septal leaflet of the tricuspid valve, which results in discontinuity of the central fibrous body and septal atrioventricular ring leading to direct muscular connections.^4^

Wolff-Parkinson-White is a pre-excitation syndrome caused by an accessory pathway, which can be associated with SVT. The term ‘WPW syndrome’ is reserved for patients with typical ECG findings of ventricular pre-excitation, including a short PR interval and a broad QRS complex with a slurred upstroke, in addition to symptomatic tachyarrhythmia. In the absence of tachycardia, the ECG findings noted above are termed ‘WPW pattern’. Recurrent SVT can result in tachycardia-induced cardiomyopathy, however there is a proportion of patients who develop a dilated cardiomyopathy in the absence of documented SVT.^5–8^ In patients with Wolff-Parkinson-White, there is pre-systolic depolarisation and contraction of specific myocardial segments dependent on the location of the accessory pathway. The development of dilated cardiomyopathy is influenced by the location of the accessory pathway and is more likely to be seen with right-sided septal pathways. These pathways result in abnormalities of septal wall motion, which can lead to adverse remodelling of the left ventricle and progressive dilatation and dysfunction as seen in our patient. Similar abnormalities of septal wall motion are also seen in patients with left bundle branch block. Loss of ventricular pre-excitation can result in an acute increase in left ventricular systolic function followed by reverse remodelling and complete recovery of left ventricular function over the course of weeks.^5–8^

Electrophysiology study and ablation provides a definitive management strategy for patients with accessory pathway-mediated left ventricular dysfunction; however, the risk profile of this procedure is increased in patients <15 kg.^9^ Current guidelines recommend reserving catheter ablation in the smallest children (infants weighing 3–7 kg and <6 months of age) for those with life threatening arrhythmias or refractory arrhythmias after multiple failed attempts at medical management.^9^ In those in whom an electrophysiology study and ablation carries higher risk, medical management with flecainide can be used to block antegrade conduction via the accessory pathway to achieve resynchronisation and normalisation of ventricular function. Flecainide is a class Ic antiarrhythmic agent, which acts by selectively blocking cardiac fast inward sodium current resulting in slowed conduction and increased refractory periods.^10^ Based on results from the cardiac arrhythmia suppression trial, flecainide is not recommended for use in patients with structural heart disease (heart failure/myocardial ischaemia) due to an increased risk of ventricular arrhythmia and mortality.^11^ These findings raised concerns for its use in paediatric patients with congenital heart disease (CHD) or cardiomyopathy, however safe use of flecainide has been demonstrated in this population.^12^ This report also illustrates the safe use of flecainide in an infant with CHD with no reported adverse effects. We monitored for evidence of toxicity using serial ECGs to measure QRS duration as well as measurement of a flecainide trough level 7 days after commencement. In addition, this infant underwent Holter monitoring to detect any intermittent WPW pattern or sub-clinical arrhythmia.

Finally, other causes for left ventricular dysfunction in Ebstein anomaly were considered. Abnormalities of left ventricular morphology and function have been associated with Ebstein anomaly with one series demonstrating left ventricular non-compaction in 18% of patients with Ebstein anomaly.^13^ Furthermore, causative mutations in sarcomere genes such as MYH7 and TPM1 have been described in patients with left ventricular non-compaction in conjunction with Ebstein anomaly.^14,15^ In older patients, ventricular interdependence between the dilated right ventricle and smaller left ventricle likely plays a role in abnormalities of left ventricular function which has been correlated with functional exercise capacity.^16^

This case highlights the known association between Ebstein’s anomaly and WPW pattern as well as the utility of flecainide in blocking antegrade conduction via the accessory pathway to achieve ventricular synchrony.

## Lead author biography


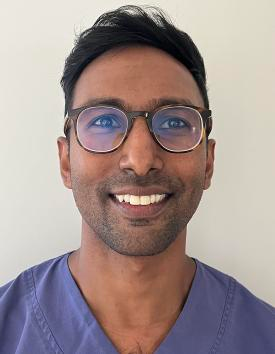


Dr Gajon Uthayakumaran is a final year advanced trainee in Paediatric Cardiology at the Children's Hospital Westmead, Sydney. He is pursuing subspecialty training in paediatric interventional cardiology.

## Supplementary Material

ytae442_Supplementary_Data

## Data Availability

The data underlying this article are available in the article and in its online Supplementary material.
